# FGFR3 has tumor suppressor properties in cells with epithelial phenotype

**DOI:** 10.1186/1476-4598-12-83

**Published:** 2013-07-31

**Authors:** Marie Lafitte, Isabelle Moranvillier, Stéphane Garcia, Evelyne Peuchant, Juan Iovanna, Benoit Rousseau, Pierre Dubus, Véronique Guyonnet-Dupérat, Geneviève Belleannée, Jeanne Ramos, Aurélie Bedel, Hubert de Verneuil, François Moreau-Gaudry, Sandrine Dabernat

**Affiliations:** 1 INSERM U1035, Université Bordeaux Segalen, 146 rue Léo Saignat, Bordeaux 33076, France; 2 Plate-forme de vectorologie, Université Bordeaux Segalen, Bordeaux 33076, France; 3 INSERM U1068, Marseille 13288, France; 4 Animalerie A2, Université Bordeaux Segalen, Bordeaux 33076, France; 5 EA2406 Université Bordeaux Segalen, Bordeaux 33076, France; 6 CHU de Bordeaux, Bordeaux 33051, France; 7 Tumorothèque de Montpellier, Montpellier 34295, France

**Keywords:** FGFR3, Pancreatic cancer, Tumor suppressor, Oncogene, MAP kinases, STAT

## Abstract

**Background:**

Due to frequent mutations in certain cancers, *FGFR3* gene is considered as an oncogene. However, in some normal tissues, *FGFR3* can limit cell growth and promote cell differentiation. Thus, *FGFR3* action appears paradoxical.

**Results:**

FGFR3 expression was forced in pancreatic cell lines. The receptor exerted dual effects: it suppressed tumor growth in pancreatic epithelial-like cells and had oncogenic properties in pancreatic mesenchymal-like cells. Distinct exclusive pathways were activated, STATs in epithelial-like cells and MAP Kinases in mesenchymal-like cells. Both *FGFR3* splice variants had similar effects and used the same intracellular signaling. In human pancreatic carcinoma tissues, levels of FGFR3 dropped in tumors.

**Conclusion:**

In tumors from epithelial origin, *FGFR3* signal can limit tumor growth, explaining why the 4p16.3 locus bearing *FGFR3* is frequently lost and why activating mutations of *FGFR3* in benign or low grade tumors of epithelial origin are associated with good prognosis. The new hypothesis that FGFR3 can harbor both tumor suppressive and oncogenic properties is crucial in the context of targeted therapies involving specific tyrosine kinase inhibitors (TKIs). TKIs against FGFR3 might result in adverse effects if used in the wrong cell context.

## Introduction

The fibroblast growth factor receptor 3 (FGFR3) belongs to the family of tyrosine kinase receptors [1]. FGFR activation induces proliferation and migration in many physiological situations, but in some cell types, FGF signaling induces differentiation and/or cell proliferation inhibition and/or cell death. Deregulation of FGF signaling in carcinogenesis has been widely explored. On one hand, it is now well-established that FGFs and FGFRs can be oncogenic through different intracellular molecular targets leading to increased cell proliferation, cell survival, angiogenesis and promotion of cell migration and invasion. On the other hand, FGF tumor suppressive effects are more and more documented, in particular for FGFR2 signaling (reviewed in [1]). In normal tissues, FGFR3 might have a negative impact on cell growth, the most striking example being its function during bone development confirmed by activating mutations in human skeletal disorders. In growth plates of the developing long bones, FGFR3 negatively controls cell proliferation and induces chondrocyte apoptosis in late embryonic stages (reviewed in [2]). In the same way, mice lacking FGFR3 receptor showed extended bone length [3] and transgenic mice with activating mutations are dwarfs [4]. In pancreatic and intestinal mouse tissues, we previously showed that FGFR3 has a negative impact on normal epithelial cell proliferation [5,6]. In cancers, activating somatic mutations of *FGFR3* were first characterized in bladder cancer and cervix cancer [7]. In bladder cancers, mutations occur preferentially in non-muscle invasive disease and much less commonly in muscle-invasive lesions, suggesting that these alterations could be linked to a favorable course of the disease in non invasive papillary bladder cancer [8]. Seborrheic keratoses and epidermal nevi, benign tumors of the skin, can also present activating mutations of *FGFR3* (reviewed in [9]). In colorectal tumors, mutations were possibly inactivating mutations, while decreased expression of FGFR3 was found in colorectal cancer cell lines [10,11] and tumors [11]. Conversely, multiple myelomas can harbor a t(4:14) intergenic translocation bringing FGFR3 gene under the control of the strong immunoglobulin heavy chain promoter, participating in tumor progression [12].

Despite contradictory results in different tumor types and models, to date, FGFR3 pathway is considered to be oncogenic in human tumors, by contrast to the situation in normal development of the long bones. Moreover, little is known on FGFR3 actions in pancreatic tumors. We explored the actions of FGFR3 in modulating pancreatic cancer cell behavior.

## Results

### FGFR3 overexpression has tumor suppressive effects

**Figure 1:**
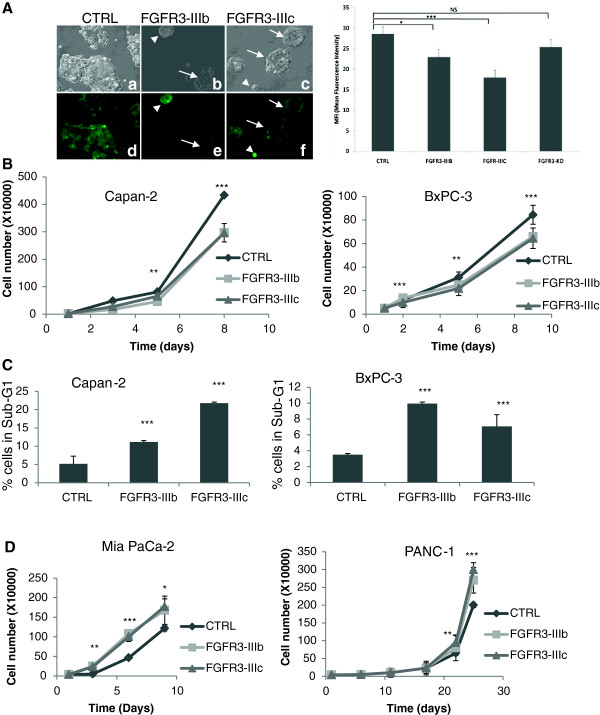
**FGFR3 has tumor suppressor gene properties in BxPC-3 and Capan-2 pancreatic cell lines. A)** Capan-2 cells were transduced with either control **(a**,**d)**, FGFR3-IIIb **(b**,**e)** or FGFR3-IIIc ZsGreen lentivectors **(c**,**f)** and observed with a fluorescence microscope (original magnification ×40, bright field, **a**,**b**,**c**, green fluorescence, **d**,**e**,). Colonies with strong overexpression of either FGFR3-IIIb or –IIIc were smaller (arrows heads, **b**, **c**, **e**, **f**) than colonies with lower expression (arrows). Right panel: mean fluorescence intensity was determined for colonies transduced with the same lentivectors as in left panel, and were also transduced with a lentivector carrying a FGFR3-IIIc cDNA with the K508M mutation inactivating the kinase domain. **B)** FGFR3-IIIb and –IIIc overexpression decreased Capan-2 and BxPC-3 cell proliferation and **(C)** increased apoptosis. **(D)** FGFR3 forced expression had opposite effect on cell proliferation of Mia PaCa-2 and PANC-1 cells. NS: nor significant, *p = 0.05, **p < 0.01, ***p < 0.001.

*FGFR3* is expressed as two splice variants, the *FGFR3-IIIb* and *FGFR3-IIIc*. Forced expression of either splice variants was performed in two pancreatic ductal adenocarcinoma (PDAC) cell lines: the Capan-2 and the BxPC-3 pancreatic cells. The capacities of the cells to form clones in low density cultures was increased by both *FGFR3-IIIb* and *IIIc* as compared to the control condition, except for the *IIIb* splice variant in the BxPC-3 cells (Table 1). Interestingly, this effect was maintained when cells were transduced with a FGFR3-IIIc cDNA lacking tyrosine kinase activity (K508M mutation, FGFR3-IIIc-KD, Table 1). Interestingly however, the areas of the colonies were smaller with FGFR3 overexpression but not with FGFR3-IIIc-KD (Table 1). Noticeably, clones overexpressing high levels of FGFR3 appeared much smaller than clones expressing lower levels as attested by ZsGreen protein fluorescence intensity (Figure 1A, left panel). Mean fluorescence intensities were lower for cells transduced with active forms of FGFR3 (Figure 1A, right panel). Thus, *FGFR3* promoted colony formation of pancreatic cells, but clone expansion was reduced. This latter effect depended on active FGFR3 tyrosine kinase. Furthermore, forced expression of both *FGFR3* splice variants in Capan-2 and BxPC-3 cells reduced the cell proliferation (Figure 1B). Flow cytometry analyses did not evidence major differences in the distributions of cells in the cell cycles phases (Additional file 1: Figure S1), but a significant and important accumulation of cells in sub-G1 was observed, suggesting that FGFR3 induced apoptosis (Additional file 1: Figure S1 and Figure 1C).

**Table 1:** FGFR3 impact in pancreatic tumour cell lines

	**Epithelial-like**									**Mesenchymal-like**						
			***Capan-2***				***BxPC-3***				***Mia PaCa-2***				***PANC-1***	
	**Colony formation (Ratio/CT)**	**Colony area (μm**^**2**^**)**	**Migration (number of cells)**	**Senescent cells (%)**	**Tumour mass (mg)**	**Colony formation (Ratio/CT)**	**Colony area (mm**^**2**^**)**	**Tumour mass (mg)**	**Colony formation (Ratio/CT)**	**Colony area (mm**^**2**^**)**	**Migration (number of cells)**	**Senescent cells (%)**	**Tumour mass (mg)**	**Colony formation (Ratio/CT)**	**Colony area (mm**^**2**^**)**	**Tumour mass (mg)**
**CTRL**	1 ± 0.13	19.3 ± 5.0	997 ± 6	24.4 ± 5.3	203 ± 37	1 ± 0.18	24.2 ± 15.1	42 ± 0.01	1 ± 0.004	13.8 ± 4.7	98 ± 2	8.6 ± 1.9	21 ± 3	1 ± 0.05	9.9 ± 9.2	59 ± 39
**FGFR3-IIIb**	1.38 ± 0.06*	9.1 ± 1.5*	1524 ± 26***	35.7 ± 3.9**	110 ± 12*	0.97 ± 0.10	15.9 ± 11.9**	25 ±0.01*	1.52 ± 0.18**	17.0 ± 4.6**	346 ± 9***	4.7 ± 1.9***	292 ± 133*	1.28 ± 0.04*	21.5 ± 14.1***	137 ± 51*
**FGFR3-IIIc**	1.52 ± 0.27*	6.3 ± 1.5**	2050 ± 18***	32.4 ± 5.5*	111 ± 14*	1.37 ± 0.04*	12.7 ± 9.7***	23 ± 0.01*	1.28 ± 0.17*	23.7 ± 8.1***	515 ± 22***	5.0 ± 1***	367 ± 113**	1.23 ± 0.04*	22.8 ± 14.6***	138 ± 39*
**FGFR3-IIIc-KD**	1.21 ± 0.05*	15.7 ± 11.1	1492 ± 10***	16.9 ± 3.26*	212 ± 71	1.26 ± 0.04**	20.9 ± 14.2	ND	ND	ND	ND	ND	ND	1.55 ± 0.12*	15.1 ± 8.7**	ND

Cells were transduced to obtain 70% of transduced cells as analyzed by flow cytometry. For colony formation assays, 500 cells were plated in 10 mm dished in triplicates. Dishes were processed for violet crystal staining and blind counted. Colony areas were measured with Image J software. Senescence was detected as indicated in the methods section. Senescence was not detectable in BxPC-3 and PANC-1 cells. Results are reported as means ± SD except for tumour masses ± SEM. *: p < 0.05 **: p < 0.01 ***: p < 0.001 as compared to control. Ratio/CT: results are expressed as ratio over the control condition. ND: not done.

Over time Capan-2 cells that appeared viable were still detectable by light microscopy. Viable cells stopping proliferation, but not proceeding to apoptosis, could be undergoing senescence. Acidic beta-galactosidase staining associated with the senescent phenotype in numerous studies (reviewed in [13]). It was significantly increased in *FGFR3-IIIb* and *-IIIc* overexpressing cells compared with the controls (Table 1, and Additional file 2: Figure S2A) in Capan-2 cells, but not in BxPC-3 cells where senescence did not seem to occur at all.

To further test whether the effects of *FGFR3* seen *in vitro* were valid *in vivo*, transduced cells, sorted by flow cytometry were xenografted in immune-compromised mice and tumor progression was examined. Very interestingly, overexpression of both *FGFR3-IIIb* and –*IIIc* inhibited Capan-2 and BxPC-3 tumor growth (about 2-fold decrease, Table 1). Noticeably, establishment of single clones overexpressing *FGFR3-IIIc* in Capan-2 cells showed that tumor growth inhibition was dependent on *FGFR3-IIIc* expression levels (R^2^ about 0.87, Additional file 3: Figure S3). The tumor masses were not different from controls when Capan-2 cells overexpressed FGFR3-IIIc-KD, suggesting that the negative impact on tumor progression depended on FGFR3 tyrosine kinase activity (Table 1).

### FGFR3 impacts pancreatic epithelial- and mesenchymal-like cells differently

*FGFR3* behaved as a tumor suppressor in pancreatic cells, which was contradictory with its proposed oncogenic properties described in the literature. To further confirm this surprising result, both splice variants were overexpressed in two additional pancreatic adenocarcinoma cell lines. As observed for the two previous cell lines, colony formation was induced by *FGFR3* in both the Mia PaCa-2 and PANC-1 cells, independently of FGFR3-IIIc kinase activity for the PANC-1 cells (Table 1). However, this time, colony size was unexpectedly significantly enhanced even when the FGFR3-IIIc lacked kinase activity (Table 1). This feature was in agreement with increased proliferation (Figure 1D). In Mia Paca-2 cells, cell cycle analysis by flow cytometry showed that cells in S phase were about 2-fold more numerous in the presence of FGFR3 forced expression (Additional file 1: Figure S1). Senescence was not detected in PANC-1 cells and was low but decreased in Mia PaCa-2 cells (Table 1 and Additional file 2: Figure S2B). *In vivo* tumor formation confirmed the *in vitro* data since overexpression of both *FGFR3-IIIb* and –*IIIc* strongly promoted PANC-1 (2-3-fold increase, Table 1) and even more the Mia PaCa-2 (13-17-fold increase, Table 1) tumor growth.

It seemed that FGFR3 impacted cancer cell clonogenic properties independently of its kinase activity. We further tested Capan-2 cell migration capacities. Again cell migration was increased by FGFR3, independently of its kinase activity (Table 1).

**Figure 2:**
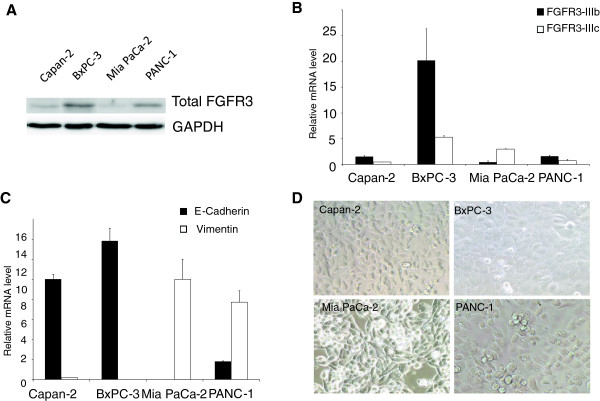
**Phenotypic properties of PDAC cell lines. A)** FGFR3 expression in the PDAC cell lines was determined by western-blot. FGFR3 expression was the highest in BxPC-3 cells and the lowest in Mia PaCa-2 cells. Membranes were reprobed for GAPDH to test equivalent loading. **B)** FGFR3 splice variants relative mRNA level were determined by RT-qPCR with specific primers and normalized with RPLP0 used as a mRNA expression internal reference. **C)** Epithelial E-cadherin and mesenchymal vimentin markers mRNA levels were determined by RT-qPCR. Capan-2 and BxPC-3 cells were epithelial, whereas the Mia PaCa-2 cell line was more mesenchymal. PANC-1 cells displayed an intermediate phenotype. **D)** Light microscope pictures of the four tested cell lines (original magnification ×40). PANC-1 presented an epithelial phenotype despite the strong expression of vimentin.

The different responses of the cells could be linked to various endogenous FGFR3 levels, before over-expression. Western-blots showed that BxPC-3 cells expressed the highest levels of the receptor, and the Mia PaCa-2 cells the lowest (Figure 2A). The two other cell lines displayed similar intermediate FGFR3 protein levels, but opposite behavior in response to *FGFR3* forced expression. RT-qPCRs further showed that *FGFR3-IIIb* isoform was more abundant, except for the Mia PaCa-2 cells (Figure 2B). In addition total FGFR3 was detected by immunofluorescence in the cell lines, and we noticed that it had nuclear localization in PANC-1 and MiaPaCa-2 cells, but not in Capan-2 or BxPC-3 cells (Additional file 4: Figure S4). In Normal Human Keratinocytes (NHEK), signal was detected throughout the whole cells. Thus total FGFR3 levels or distinct *FGFR3* splice variant relative abundance could not explain the opposite phenotypes.

*FGFR-IIIb* isoform is preferentially expressed in cells of epithelial origin and *FGFR-IIIc* isoforms in mesenchymal cells [14]. RT-qPCRs were performed to assess the levels of expression of E-cadherin and vimentin, markers of epithelial and mesenchymal cells, respectively (Figure 2C). Capan-2 and BxPC-3 cells displayed epithelial characteristics (strong E-cadherin expression, almost no vimentin expression). The Mia PaCa-2 line was mesenchymal-like (no E-cadherin expression and strong vimentin expression) and PANC-1 s displayed an intermediate phenotype since they still expressed some E-cadherin but did express high levels of vimentin. These cells retained characteristics of epithelial cells in terms of cell shape, like the Capan-2 and BxPC-3 cells, while the Mia PaCa-2 cells resembled fibroblasts (Figure 2D). Thus, our experimental cell models were distinguishable in three categories: the epithelial-like cells (BxPC-3 and Capan-2), the mesenchymal-like cells (Mia PaCa-2) and cells with intermediate phenotype (PANC-1).

In light of this data, it appeared that in epithelial-like pancreatic cells (BxPC-3 and Capan-2), FGFR3 had tumor suppressive effects, while in cells with mesenchymal cell features (Mia PaCa-2 and PANC-1), FGFR3 had oncogenic effects.

**Figure 3:**
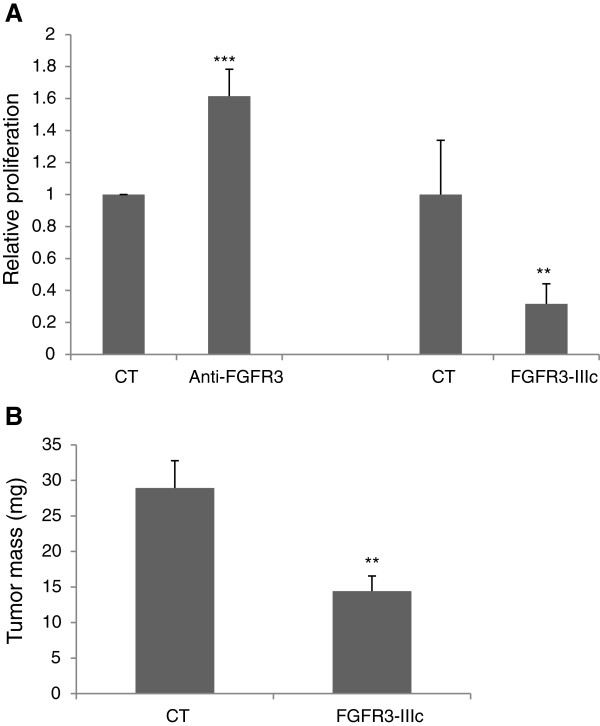
**FGFR3 conveys a negative signal in the pancreatic beta cell line BTC. A)** BTC cells were cultured in the absence (CT) or in the presence of the neutralizing anti-FGFR3. Alternatively, cells were transiently transfected with a vector overexpressing FGFR3-IIIc or a control vector (CT). **B)** Tumors were produced from parental BTC cells (n = 12) or clones of BTC cells overexpressing stably FGFR3-IIIc (n = 17). Tumor masses were determined 6 weeks after cells were injected. ***: p < 0.001, **: p < 0.01.

To further explore the impact of FGFR3 in epithelial cells, the proliferation of the mouse pancreatic epithelial beta cell line BTC [15] was assessed in the presence of a neutralizing anti-FGFR3 antibody or with FGFR3-IIIc splice overexpression *in vitro*. The inhibition of FGFR3 activity increased BTC cell proliferation, while transient forced expression of FGFR3-IIIc significantly diminished BTC cell expansion (Figure 3A). When BTC cells overexpressing FGFR3-IIIc were xenografted in immune deficient mice, tumor progression was strongly inhibited as compared to control cells (Figure 3B and Additional file 5: Figure S5). This suggests that as in pancreatic adenocarcinoma cell lines with epithelial phenotype, FGFR3 limits beta cell growth.

### FGFR3 ligands are differentially expressed in pancreatic cell lines

The different responses of the cell lines to FGFR3 signal could be related to distinct status in available FGFs. We performed all the *in vitro* experiments in complete media, without modulating the levels of FGFs. The reason was that all the PDAC cell lines expressed endogenous FGFs that could bind either splice variants of the receptor (Additional file 6: Figure S6A). Indeed, RT-qPCR aimed to evaluate the relative levels of known ligands for FGFR3 in the parental cells showed that FGF4 and FGF23 were not detectable. CAPAN2 cells expressed FGF17 and FGF18, also present in the MIAPACA2 cells at similar levels. Interestingly however, distinct profiles were obtained for other FGFs. Strikingly, MIAPACA2 and PANC1 cells expressed very high levels of FGF2 mRNAs, which were almost not detectable in the epithelial-like cells. Moreover, the CAPAN2 cells expressed FGF9, which was also detected at a lower level in BXPC3s, but not the two other cell lines. Importantly, FGF2 and FGF9 levels were increased in cells overexpressing FGFR3 (Additional file 6: Figure S6B), suggesting that autocrine loops in parental cell lines are reinforced with FGFR3 forced expression.

Thus it seems that distinct autocrine loops are present in the PDAC cell lines for FGFR3. FGF2 was clearly associated with the MIAPACA2 and the PANC1 cells, but not cells with an epithelial phenotype.

### FGFR3 signaling is different in pancreatic epithelial and mesenchymal-like cells

**Figure 4:**
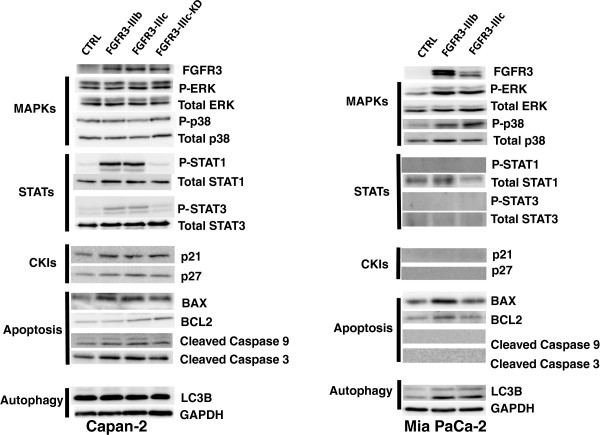
**FGFR3 engaged diverse intracellular pathways.** Proteins extracts of tumors from Capan-2 and Mia PaCa-2, were analyzed by western-blot. See text for more details. CKIs: Cyclin-dependant kinase inhibitors. Membranes were reprobed for GAPDH to test equivalent loading. Results shown are representative of one out of at least 3 independent experiments.

In the presence of the ligand, the dimerization of the receptor triggers FGFR transphosphorylations, to create docking sites for downstream signaling factors activating pathways such as RAS and the downstream MAP Kinases, the PI3Kinase/Akt pathway or the Signal transducer and activator of Transcription (STAT) pathway. The protein extracts of tumors overexpressing FGFR3 with the strongest and opposite phenotype*s* (Capan-2 and Mia PaCa-2 cells) were analyzed by western-blotting. Active JNKs, detectable only in Capan-2 cells, were unchanged (not shown). In FGFR3-Capan-2 cells, other activated MAP Kinases were unchanged (Figure 4), but in Mia PaCa-2 cells both ERKs and P38 were over-activated (Figure 4). Conversely, phosphorylation of STATs was found strongly increased by FGFR3 overexpression in Capan-2 cells but was not detected in Mia PaCa-2 cells. This effect in Capan-2 cells disappeared when transduced with FGFR3-IIIc-KD. Although AKT was present in all the tumor extracts, no changes were observed in activated AKT (not shown). The proteins p21 and p27, downstream effectors of FGFR3 in chondrocytes [2] were mildly increased in FGFR3-Capan-2 tumors (Figure 3), but not in the Mia PaCa-2 (not detectable). Since apoptosis was found up-regulated in FGFR3-Capan-2 cells, we looked at apoptosis markers. BAX signal was stronger in FGFR3 conditions, while BCL2 remained unchanged (Figure 4). Thus the balance of anti-apoptotic/pro-apoptotic proteins was moved towards apoptosis. This was further confirmed by increased levels of both cleaved caspase 9 and caspase 3 (Figure 4). In Mia PaCa-2 s, there was no obvious change in the anti-apoptotic/pro-apoptotic balance and cleaved caspases were not detectable (Figure 4). Senescence often depends on p53 activation or stabilization. The Capan-2 cells were the only cell line in our hands bearing wild type copies of *TP53*. Total p53 and Phospho-p53 were not changed in FGFR3 overexpressing cells (not shown).

Finally, autophagy can promote proliferation or apoptosis. LC3B protein conversion status was evaluated. Both LC3B-I and LC3B-II peptides were detected, indicating that autophagy is active in Capan-2 cells. However, FGFR3 did not induce any change in LC3B-I cleavage (band intensity of LC3B-II/LC3B-I, Figure 4B). In Mia PaCa-2 cells, the basal levels of both LC3B forms were increased in the presence of FGFR3 overexpression, together with the rate of LC3B-I cleavage (Figure 4). This suggests that FGFR3 promoted autophagy in the Mia PaCa-2 cells.

Thus, it seemed that in epithelial-like cells FGFR3 activated STATs but not ERKs and in mesenchymal cells, it was the opposite. To further explore this point, western-blots were performed with tumor extracts from BxPC-3 and PANC-1 cells. As in Capan-2 cells, BxPC-3 cells showed increased STATs activation and unchanged P-ERK levels (Additional file 7: Figure S7). Surprisingly, in PANC-1, none of the tested signaling seemed affected by FGFR3 forced expression, despite phenotypes similar to what was observed in the MiaPaCa-2 cells (Additional file 7: Figure S7). P-ERKs and P-JNKs were evidenced but not modulated and P-p38 was undetectable even if p38 was present (Additional file 7: Figure S7). In the same way although P-STAT1 proteins were detected in the PANC-1 cell extracts, they were not induced by either FGFR3 splice variant. BAX and Bcl-2 were unchanged. Thus, in PANC-1 cells, the oncogenic effect of FGFR3 was evidenced, but it clearly depended on intracellular signals different from those in the Capan-2 or the MiaPaCa-2 cells. Cell proliferation capacities are strongly modulated by phosphorylation of RB1 protein. This protein is detected and active in many PDAC cell lines, including the ones used in the present report [16]. RB1 phosphorylation at serine 795 was induced in PANC1 cells overexpressing FGFR3 but not in Capan-2 cells where it was detectable or MIAPACA2 cells where it was undetectable, suggesting that RB1 activity was diminished in PANC1 cells (Additional file 7: Figure S7). In the same way, CREB phosphorylation was induced in PANC1 cells and was undetectable in CAPAN2 cells or in MIAPACA2 cells.

Thus, FGFR3 intracellular partners were the same in the epithelial-like cancer cells, but different in the mesenchymal-like cells.

### FGFR3 expression is downregulated in human pancreatic tumor tissues

To gain insight into the impact of FGFR3 signalling pathway specifically in pancreatic cancers, pancreatic cancer tissues were analyzed for the levels of expression of the receptor compared to normal pancreatic tissue.

**Figure 5:**
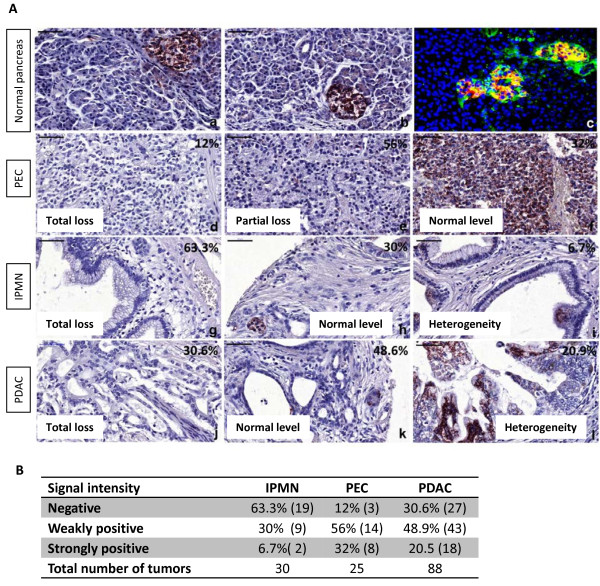
**FGFR3 expression in normal and pancreatic tumor tissues. A: (a**, **b)** FGFR3 signal was found in islets of normal pancreas. **(c)** Co-localization of FGFR3 (green) and insulin (red). Nuclei were stained with DAPI (blue). **(d**-**l)** FGFR3 expression was assessed according to the signal obtained in normal pancreas (strong in islets, weak in the exocrine tissue). Twelve percent of pancreatic endocrine carcinomas (PEC, n = 25) displayed no FGFR3 signal **(d)**, 56% showed weak **(e)** and only 32% had strong (normal, **f**) FGFR3 expression. For Intraductal papillary mucinous neoplasms (IPMN, n = 30), 63.3% had no signal **(g)**, 30% had weak signal (normal, **h**) and 6.7% showed strong **(i)** FGFR3 expression. In Pancreatic ductal adenocarcinomas (PDAC, n = 88), 30.6% displayed no FGFR3 signal **(j)**, 48.9% had weak (normal, **k**) and 20.5% showed strong **(l)** FGFR3 expression. Bars represent 50 μm. **B:** Table recapitulating the scoring of pancreatic tumors for FGFR3 signal. Strong signals were as intense as signals obtained in normal pancreatic islets **(A**: **a**, **b)**. Weak signals were similar to what was observed in normal pancreatic exocrine tissue (ducts and acinar cells, **A: a**, **b**).

Strong FGFR3 immune signal was found in normal pancreatic islets, while a weak signal was present in the exocrine tissue (Figure 5a,b). FGFR3 was co-localized with insulin-positive cells, but was also present in non beta cells in islets (Figure 5c). Both splice variants appeared to be equally abundant in the normal pancreas (Additional file 8: Table S1).

In cancer tissues, we found a significant decrease of FGFR3 RNAs in PDAC as compared to normal pancreas, regardless of the splice variant, which balance did not change (Additional file 8: Table S1). In tissue microarrays, when compared to FGFR3 signal intensity in normal pancreas (high in islets, weak in ducts), about 70% of the pancreatic endocrine carcinomas showed partial or total loss of FGFR3 signal (Figure 5d-f). 63% of the intraductal papillary mucinous neoplasms and 30% of the pancreatic ductal adenocarcinomas showed total loss of FGFR3 signal. Some of the two latter cancer tissues displayed areas with strong staining, but it was always highly heterogeneous (Figure 5g-l), some regions totally lacking FGFR3, on the same sections (Figure 5i and 5l). Thus, *FGFR3* transcripts and protein seemed to be lost in a non negligible proportion of pancreatic tumors, especially in endocrine tumors.

## Discussion

### *The impact of FGFR3* mutations in various tumors can be revisited in light of the present study

Early published data converged towards oncogenic properties of *FGFR3*. Indeed, in multiple myelomas, the chromosomal translocation t(4;14)(p16.3;q32) results in ectopic overexpression of *FGFR3 *[12]. Further, *FGFR3* was considered as an oncogene in hematopoietic cell models [17] and in 3T3 fibroblast, but not in the T24 epithelial cells [18]. In other soft tissue tumors such as synovial carcinomas or rhabdomyosarcomas, *FGFR3* was involved in the malignant phenotype [19,20]. Results in Mia PaCa-2 cells (this work) and SW480 [11] support the notion that in tumors originating from mesenchymal cells, *FGFR3* is an oncogene.

However, puzzling observations were previously unexplained and even incompatible with *FGFR3* being an oncogene. First, the receptor has a negative regulatory role during bone development. In colorectal cancers, a group noticed frequent inactivation of the receptor or downregulation of the protein [10]. Importantly, frequent loss of heterozygosity of the chromosomal region carrying *FGFR3* gene (the 4p16.3 locus) has been characterized, suggesting that this region carries a tumor suppressor gene [21]. In that context, it was surprising to find *FGFR3* as an oncogene in this locus. *FGFR3* expression was reduced in almost 60% of cervix tumors and when it was increased, it was in tumors of good prognosis [22]. Later on, critical data showed a significant association of loss of *FGFR3* and tumor stage in high grade urothelial bladder cancers [23]. Accordingly, *FGFR3* activating mutations were associated with early, non invasive, low grade papillary tumors and never found in carcinomas *in situ *[24]. Consequently, active mutants of *FGFR3* are now associated with low grade tumors in urothelial cancers and possible loss of heterozygosis at *FGFR3* locus might accompany the switch to higher grades (reviewed in [25]). In the same way, in seborrheic keratoses and epidermal nevi, benign tumors of the skin very rarely progressing into malignant disease, also present high rates of activating mutations of *FGFR3* (reviewed in [9]), suggesting a protective role of increased *FGFR3* in these models, of epithelial origin. Our data support the concept that *FGFR3* signal in epithelial context might actively participate to limit loss of proliferation control, and inhibit the switch to malignant disease. In pancreatic cancer, this hypothesis is in agreement with the loss of expression found in RNA and tissue sections of pancreatic cancers (the present work). It would be very interesting to check the status of cell phenotypes in the same tumors to determine the proportion of lesions which have undergone the epithelial to mesenchymal transition. We did not have the clinical parameters of the patients from whom the lesions came from. Additional experiments are needed to determine whether loss of FGFR3 signal correlates with bad prognosis of the disease. Moreover, it would be interesting to test the possibility that pancreatic lesions with mesenchymal phenotype retain *FGFR3* expression. In colorectal cancer models [11], authors could not explain the loss of *FGFR3* expression in human tumors since they believed that FGFR3 was an oncogene. However, they found, like us, a downregulation of *FGFR3* expression in colorectal cancers. They used several colorectal cancer cell lines to evaluate *FGFR3* impact on growth and migration. They could not obtain *FGFR3* transfectants with the CaCo2 cell line and observed the HCT116 cells being inhibited by *FGFR3* overexpression. Opposite phenotype was reported in the SW480 cells, and the main conclusions of the report were drawn based on this cell line only. Interestingly, HCT116 cells express high levels of E-cadherin and low levels of vimentin [26], while SW480 cells do not express E-Cadherin [27], but vimentin [28]. It is possible that CaCo2 cells died in the presence of FGFR3 forced expression. We ourselves encountered difficulties in obtaining single Capan-2 or BTC clones with FGFR3-IIIc overexpression. Thus the conclusions of Sonvilla et al. might be reformulated in light of our results.

### Molecular mechanisms of FGFR3 signals in PDAC cells

*FGFR3* oncogenic effect seemed to be conveyed by MAP kinases activation in the Mia PaCa-2 cells, even if several studies report that KRAS is mutated in that cell line, as in most PDAC cells. It means that an alternative pathway transmitted FGFR3 signal in those cells to over-activate MAP kinases. This activation but not that of STATs is similar to what was found in multiple myelomas [29]. The differences in percentages found in flow cytometry analyses could not explain the differences in cell counts. It is possible that the higher rate of autophagy contributes to cell growth promotion in these cells, as suggested by variations in LC3B levels and cleavage rate. This hypothesis needs further analysis in future. Interestingly, in PANC-1 cells FGFR3 had oncogenic properties but signaling was different to what was evidenced in Mia PaCa-2s. Alternative pathways might occur depending on intracellular available adaptors and lead to phenotypes with different intensities as observed in this study.

Distinct autocrine loops were evidenced in PDAC cells. It is possible that opposite effects were FGF-dependent (FGF2 for the oncogenic effect and FGF9 for the tumor suppressive effect), the role of FGF2 being supported by previous studies ([30] for example) but more work is needed to test the dependency of FGFR3 to FGFs.

The most relevant finding of this study is the tumor suppressive properties of FGFR3 in pancreatic ductal adenocarcinoma epithelial cells. In the growth plates of developing long bones, *FGFR3* negatively controls cell proliferation and induces chondrocyte apoptosis in late embryonic stages, noticeably through STATs activation (reviewed in [2]). This, together with induction of senescence could account for the loss of cell numbers observed in our proliferation assays. It has been shown recently that *FGFR3-IIIc* can induce premature reversible senescence in chondrocytes [31]. Authors found, like us, that p53 was active in their cells but that FGFR3-induced senescence was independent of p53. In our hands, the main difference between the epithelial-like and the mesenchymal-like cell response to FGFR3 signal was the activation of STATs, which can elicit senescence response independent of p53 [32]. Since we found p21 modestly induced in FGFR3 overexpressing cells, it is possible that activated STATs directly controlled *CDKN1A* gene promoter, as earlier described [33].

**Figure 6:**
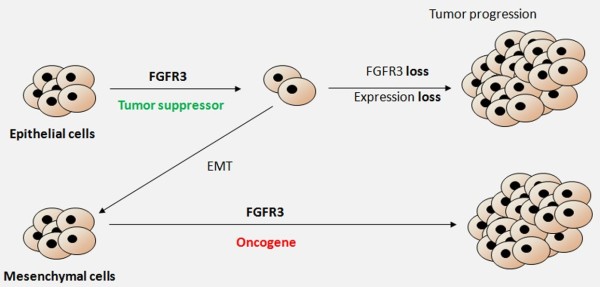
**FGFR3 actions in pancreatic cancers: a working model.** FGFR3 action in cancer cells from epithelial origin limits tumor growth. During tumor progression, FGFR3 disruption or loss of expression promotes cell growth. If epithelial to mesenchymal transition occurs, then FGFR3 will function as an oncogene favoring tumor progression.

The present data raise the possibility that *FGFR3* has biphasic effects during multistage carcinogenesis in carcinomas, acting first as a tumor suppressor through oncogene-induced senescence via STATs activation and apoptosis enhancement. In this context, FGFR3 loss would help tumor progression. Alternatively, after additional mutations have occurred in the developing tumor, decoupling FGFR3 from its canonical inhibitory pathway (STATs), FGFR3 signal might be redirected to other intracellular factors, promoting tumor progression (Figure 6). In the same way, epithelial to mesenchymal phenotype transition would reveal *FGFR3* as an oncogene by coupling the receptor to MAP kinases pathways. This model supports a new aspect of FGFR3 function, explaining why in epithelial cancers *FGFR3* activating mutations were associated with good prognosis tumors whereas in soft tissue cancers, *FGFR3* promoted tumor progression. More importantly, this work might open a new debate on the use of FGFR3 inhibitors in anticancer therapy.

## Materials and methods

### Animals, pancreatic cell lines and antibodies, tissue specimens

The 6 to 8 week-old NOD/Shi-SCID IL2R^γnull^ mice were produced and housed at the University Bordeaux Segalen animal facility A2, according to the rules and regulations of the Institutional Animal Care and Use Committee (agreement number A33063916).

The BxPC-3 and Capan-2 cells were provided by Joel Tardive-Lacombe (INSERM U624, Marseille, France). The cells were maintained in RPMI (Invitrogen, Saint Aubin, France) with 10% Fetal Calf Serum (FCS, Invitrogen) with Penicillin/Streptomycin 1/100 (Invitrogen). Mia PaCa-2 and PANC-1 cells were purchased from the ATCC (Teddington, United Kingdom) and maintained in DMEM with 10% FCS and 1/100 Penicillin/Streptomycin (Invitrogen). The BTC cells were obtained from Simon Efrat (Tel Aviv University, Israel).

The following antibodies were used: FGFR3 (SIGMA-ALDRICH, Lyon, France), Phospho-p44/p42 MAPK, p44/p42 MAPK, Phospho SAPK/JNK, SAPK/JNK, Phospho Akt, Akt, Caspase-3, Caspase-9, Phospho-STAT1, Phospho-STAT3, BAX, p21, p27, Phospho-p38, p38, GAPDH (all from Cell Signaling, Saint-Quentin en Yvelines, France).

Tissue specimens were obtained with informed consent from all patients. Tissue arrays were stained with anti-FGFR3 antibody using the Duolink® system (Eurogentec, Angers, France) with which primary antibody detection depends on a very specific PCR system, giving a punctuated brown signal.

### Vector construction and transduction of cells

The pMND-IRES2-ZsGreen1 was used as recipient vector of all the constructs. *FGFR3-IIIb* and *FGFR3-IIIc* and *FGFR3-IIIIc-K508M* were kindly provided by David Cappellen (EA2406, Univ. Bordeaux, France) and Pavel Krejci (Medical Genetics Institute, Los Angeles, California), respectively and cloned upstream the IRES sequence. *FGFR3-IIIb* and *FGFR3-IIIc* overexpressing cell lines were obtained by transduction of lentiviral plasmids. Control cells were produced by transduction of the empty pMND-IRES-ZsGreen vector. For xenografts, transduced cells were sorted by a BD FACS ARIA cell sorter (BD Biosciences, Le Pont de Claix, France).

### Isolation of RNA, cDNA synthesis, and quantitative real-time PCR analysis

Total RNA was isolated using Trizol® and treated by DNAse according to the manufacturer’s instructions (Invitrogen and Ambion, Saint Aubin, France). cDNAs were synthesized using Reverse transcriptase cDNA synthesis kit (Roche Applied Science, Meylan, France). Quantitative real-time PCR assays were performed with the SYBR® Green Master Mix and carried out on a Stratagene MX-3005P system (Stratagene, Massy, France). Sequences of primers can be provided upon request.

### Protein extraction and western-blotting

Protein extracts and western blotting were performed as already described [34].

### Cell cycle analysis

Non synchronized subconfluent cells were harvested and washed twice with PBS then fixed with 70% ethanol in PBS overnight at 4°C. Cells were washed twice with PBS and incubated with a mix containing RNAse (1 mg/ml, SIGMA-ALDRICH) and PBS-Propidium iodide (0.5 μg/ml, SIGMA-ALRICH) for 15 min. The samples were examined on a BD FACS CANTO II apparatus and the data were analyzed with BD FACSDiva software (BD Biosciences, Le Pont de Claix, France).

### SA-β-Galactosidase labeling

SA-β-galactosidase activity was observed with an inverted Nikon Microscope (Eclipse Ti Nikon, Champigny sur Marne, France). Pictures were taken with the NIS-Elements Nikon software. For SA-β-galactosidase positive cell quantification, cells were counted on twenty separate fields and the means of (100*number of blue cells/number of total cells) were calculated corresponding to the percent of SA-β-galactosidase positive cells. The numbers of counted cells were for the Capan-2: CTRL (1914), FGFR3-IIIb (2809), FGFR3-IIIc (3159), FGFR3-IIIc-KD (1687); For the MIAPAC2: CTRL (2345), FGFR3-IIIB (2718), FGFR3-IIIc (2563).

### Xenografts of PDAC cell lines

Groups of at least 5 mice were anesthetized with isoflurane. 8×10^5^ cells (4×10^5^ for BTC cells) in 100 μl serum-free medium were injected in the right flanks. When tumors were visible, measures with a calibrator were done 3 times a week. Tumors were resected and weighed at various times after the initial grafts, according to the cell line.

### Statistical analysis

*In vitro* results are expressed as mean ± SD. Results *in vivo* are expressed as mean ± SEM. Statistical tests were performed with unpaired, bilateral Student’s t tests.

## Competing interest

The authors declare no conflict of interests.

## Authors’ contributions

ML, IM and SD carried out *in vitro* experiments. Western blotting was performed by IM. *In vivo* experiments were designed by FMG, PD, EP and SD and were carried out by ML, BR, IM and SD. VGD, ML and FMG designed and produced the vectors. ML, BR, FMG and SD analyzed the results and produced the figures. SG provided the tissue array and analyzed the results with SD, ML, PD and IM. JR provided RNAs from patients and GB pancreatic tissues. EP, JI, HV and AB participated in the discussion and interpretation of the study and manuscript preparation. SD wrote the manuscript. All authors read and approved the final manuscript.

## Supplementary Material

Additional file 1: Figure S1 Flow cytometry analyses of pancreatic cell lines overexpressing FGFR3s. A: Capan-2 cells, B: BxPC-3 cells, C: MiaPaCa-2 cells. Click here for file

Additional file 2: Figure S2 SA-β-Galactosidase staining is associated with senescent cell phenotype. The staining was conducted on CAPAN2 (a, b, c) and MIAPACA2 (d, e, f) cells expressing either control, FGFR3-IIIb or –IIIc lentivectors. Senescent cells were counted in CAPAN2 cells (A, IIIb p = 0.002**; IIIc p = 0.02*) and in MIAPACA2 cells (B, IIIb p = 0.0002***; IIIc p < 10 ^-5^ ***). Black arrows: blue senescent cells. Click here for file

Additional file 3: Figure S3 FGFR3-IIIc tumor growth inhibition is dependent on FGFR3-IIIc expression levels. Single clones overexpressing FGFR3-IIIc in CAPAN-2 cells were xenografted in immuno-compromised mice. Tumors were resected and FGFR3-IIIc-overexpression level was determined by western-blot analysis of tumor-protein extracts for each clones. FGFR3-IIIc level expression of each clone was finally compared to the tumor mass. The regression was performed in Excel software. Click here for file

Additional file 4: Figure S4 FGFR3 immuno-detection in pancreatic pareantal cell lines. Parental cell lines were culture on LABTEK chambers and FGFR3 presence was detected by immunofluorescence. Normal Human Epithelial Keratinocytes (NHEK) were used as positive controls for FGFR3 presence. Original magnification ×400. Click here for file

Additional file 5: Figure S5 Western-blot of BTC cell extracts. Protein extracts from parental BTC line (lane 1) or from clones with FGFR3 overexpression (lane 2–7) have been subjected to western-blotting to detect FGFR3 and P-ERKs proteins levels. Actin protein was used as a loading control. Click here for file

Additional file 6: Figure S6 FGF expression in pancreatic cancer cell lines. A) RT-qPCRs were performed as indicated in the materials and methods section to measure the levels of expression of different FGFR3 ligands in the parental cell lines. Primers sequences can be provided upon request. B) RT-qPCRs for FGF2 and FGF9 transcripts were performed on RNA extracts from cells transduced with FGFR3-IIIb and –IIIc variants or parental cells (CTRL). Results are reported according to the levels fround in CTRL. *: p < 0.05, **: p < 0.01, ***: p < 0.001 (n = 3, as compared to CTRL levels). Note that FGF9 was not detectable in MiaPaCa-2. Click here for file

Additional file 7: Figure S7 Signaling pathways in BxPC-3 and PANC-1 tumor extracts. Proteins extracts of tumors from BxPc-3 and Mia PaCa-2, were analyzed by western-blot. See text for more details. CKIs: Cyclin-dependant kinase inhibitors. Membranes were reprobed for GAPDH to test equivalent loading. Results shown are representative of one out of at least 3 independent experiments. Click here for file

Additional file 8: Table S1 FGFR3 mRNA expression in normal pancreas and PDAC. Click here for file

## Aknowledgements

We are indebted to the Tumothèque of Bordeaux for providing us with PDAC RNA extracts. We are very grateful to Jean-Max Pasquet for his expertise on senescence analysis, to the Plateforme d’Histologie of the Université Bordeaux Segalen and to Alice Bibeyran from the Plateforme of vectorologie of Université Bordeaux Segalen. We also thank the staff from the Animaleries of the Université Bordeaux Segalen for their technical support, Pierre Costet for his support in setting up the xenograft models, and Aline Costet for her administrative help. This work was supported by grants from the Ligue contre le cancer (Comité Dordogne and Comité Landes), the Institut National du Cancer (INCa), France and funding from the SFR Transbiomed (formerly IFR66).
