# Supplementary material for: FGFR3 has tumor suppressor properties in cells with epithelial phenotype
Source: Mol Cancer. 2013 Jul 31;12:83. doi: 10.1186/1476-4598-12-83 (PMC3750311; doi:10.1186/1476-4598-12-83)
Supplement: Additional file 1: Figure S1 — Flow cytometry analyses of pancreatic cell lines overexpressing FGFR3s. A: Capan-2 cells, B: BxPC-3 cells, C: MiaPaCa-2 cells. [file 1476-4598-12-83-S1.ppt]

## Slide 1
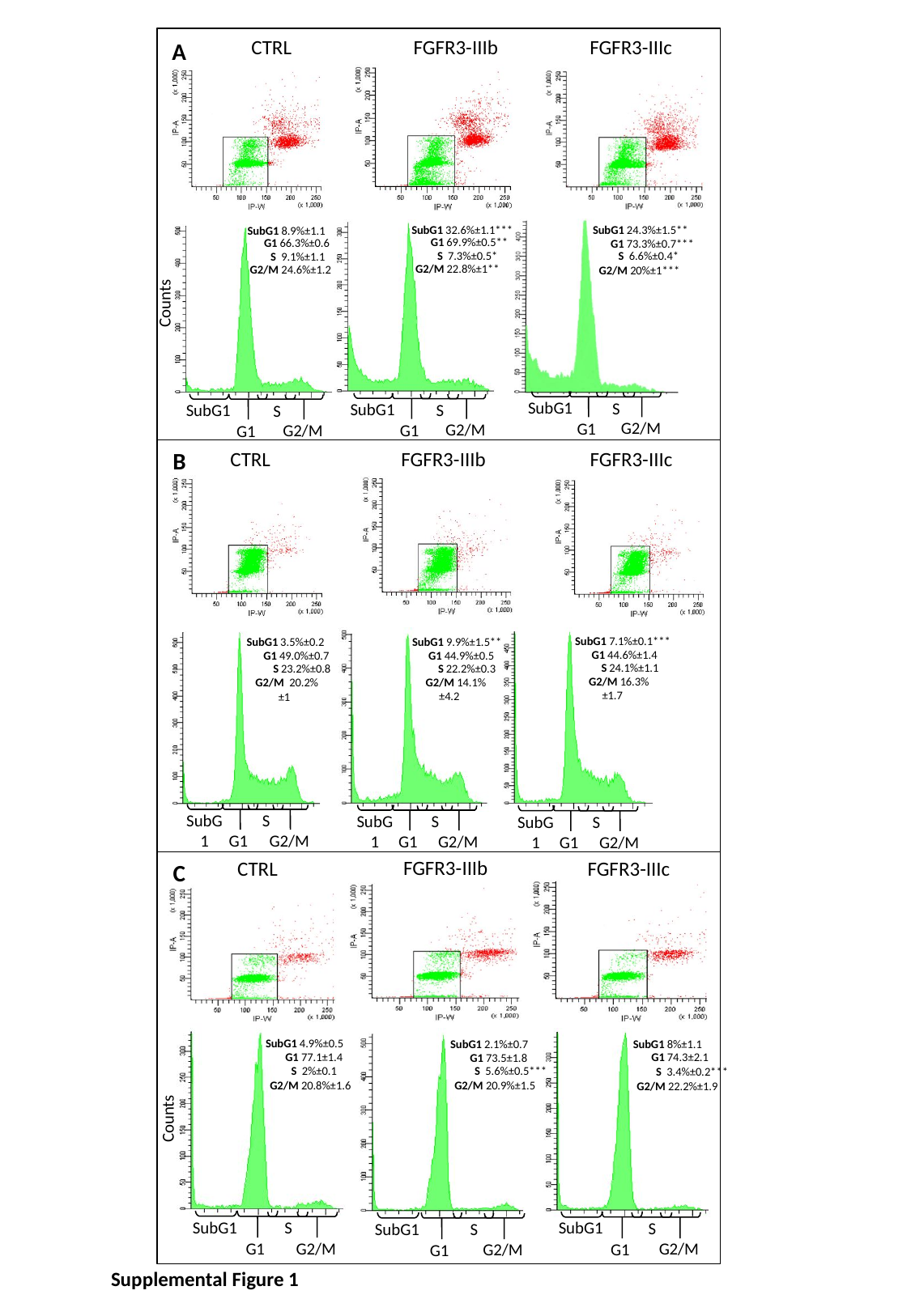

FGFR3-IIIc
CTRL
FGFR3-IIIb
A
SubG1 32.6%±1.1***
G1 69.9%±0.5**
S 7.3%±0.5*
G2/M 22.8%±1**
SubG1 24.3%±1.5**
G1 73.3%±0.7***
S 6.6%±0.4*
G2/M 20%±1***
SubG1 8.9%±1.1
G1 66.3%±0.6
S 9.1%±1.1
G2/M 24.6%±1.2
Counts
SubG1
S
G2/M
G1
SubG1
S
G2/M
G1
SubG1
S
G2/M
G1
B
CTRL
FGFR3-IIIb
FGFR3-IIIc
 SubG1 7.1%±0.1***
G1 44.6%±1.4
 S 24.1%±1.1
 G2/M 16.3%±1.7
 SubG1 9.9%±1.5**
G1 44.9%±0.5
 S 22.2%±0.3
 G2/M 14.1%±4.2
SubG1 3.5%±0.2
G1 49.0%±0.7
 S 23.2%±0.8
 G2/M 20.2%±1
SubG1
S
G2/M
G1
SubG1
S
G2/M
G1
SubG1
S
G2/M
G1
FGFR3-IIIb
FGFR3-IIIc
CTRL
SubG1 4.9%±0.5
G1 77.1±1.4
S 2%±0.1
G2/M 20.8%±1.6
SubG1 8%±1.1
G1 74.3±2.1
S 3.4%±0.2***
G2/M 22.2%±1.9
SubG1 2.1%±0.7
G1 73.5±1.8
S 5.6%±0.5***
G2/M 20.9%±1.5
Counts
SubG1
S
G2/M
G1
SubG1
S
G2/M
G1
SubG1
S
G2/M
G1
C
Supplemental Figure 1
