# Supplementary figures and images for: FGFR3 has tumor suppressor properties in cells with epithelial phenotype
Source: Mol Cancer. 2013 Jul 31;12:83. doi: 10.1186/1476-4598-12-83 (PMC3750311; doi:10.1186/1476-4598-12-83)

## Slide 1
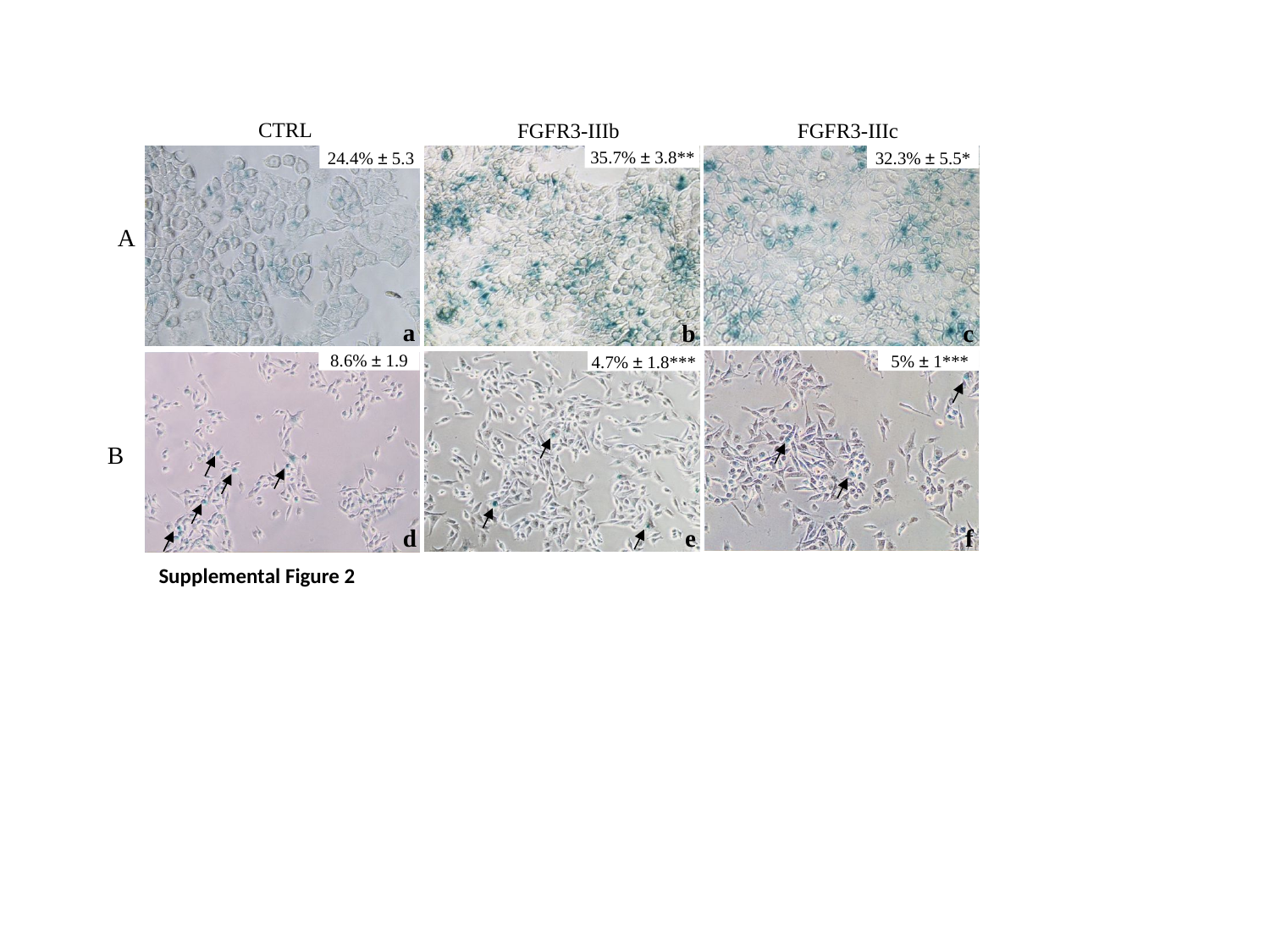

CTRL
FGFR3-IIIb
FGFR3-IIIc
35.7% ± 3.8**
32.3% ± 5.5*
24.4% ± 5.3
A
a
b
c
8.6% ± 1.9
5% ± 1***
4.7% ± 1.8***
B
d
f
e
Supplemental Figure 2

Supplement: Additional file 2: Figure S2 — SA-β-Galactosidase staining is associated with senescent cell phenotype. The staining was conducted on CAPAN2 (a, b, c) and MIAPACA2 (d, e, f) cells expressing either control, FGFR3-IIIb or –IIIc lentivectors. Senescent cells were counted in CAPAN2 cells (A, IIIb p = 0.002**; IIIc p = 0.02*) and in MIAPACA2 cells (B, IIIb p = 0.0002***; IIIc p < 10 -5 ***). Black arrows: blue senescent cells. [file 1476-4598-12-83-S2.pptx]

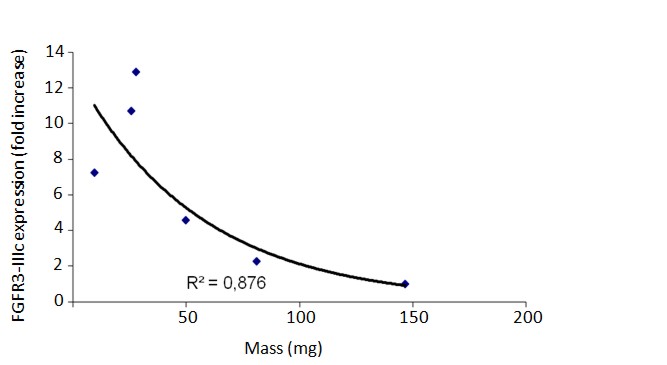

Supplement: Additional file 3: Figure S3 — FGFR3-IIIc tumor growth inhibition is dependent on FGFR3-IIIc expression levels. Single clones overexpressing FGFR3-IIIc in CAPAN-2 cells were xenografted in immuno-compromised mice. Tumors were resected and FGFR3-IIIc-overexpression level was determined by western-blot analysis of tumor-protein extracts for each clones. FGFR3-IIIc level expression of each clone was finally compared to the tumor mass. The regression was performed in Excel software. [file 1476-4598-12-83-S3.jpeg]

## Slide 1
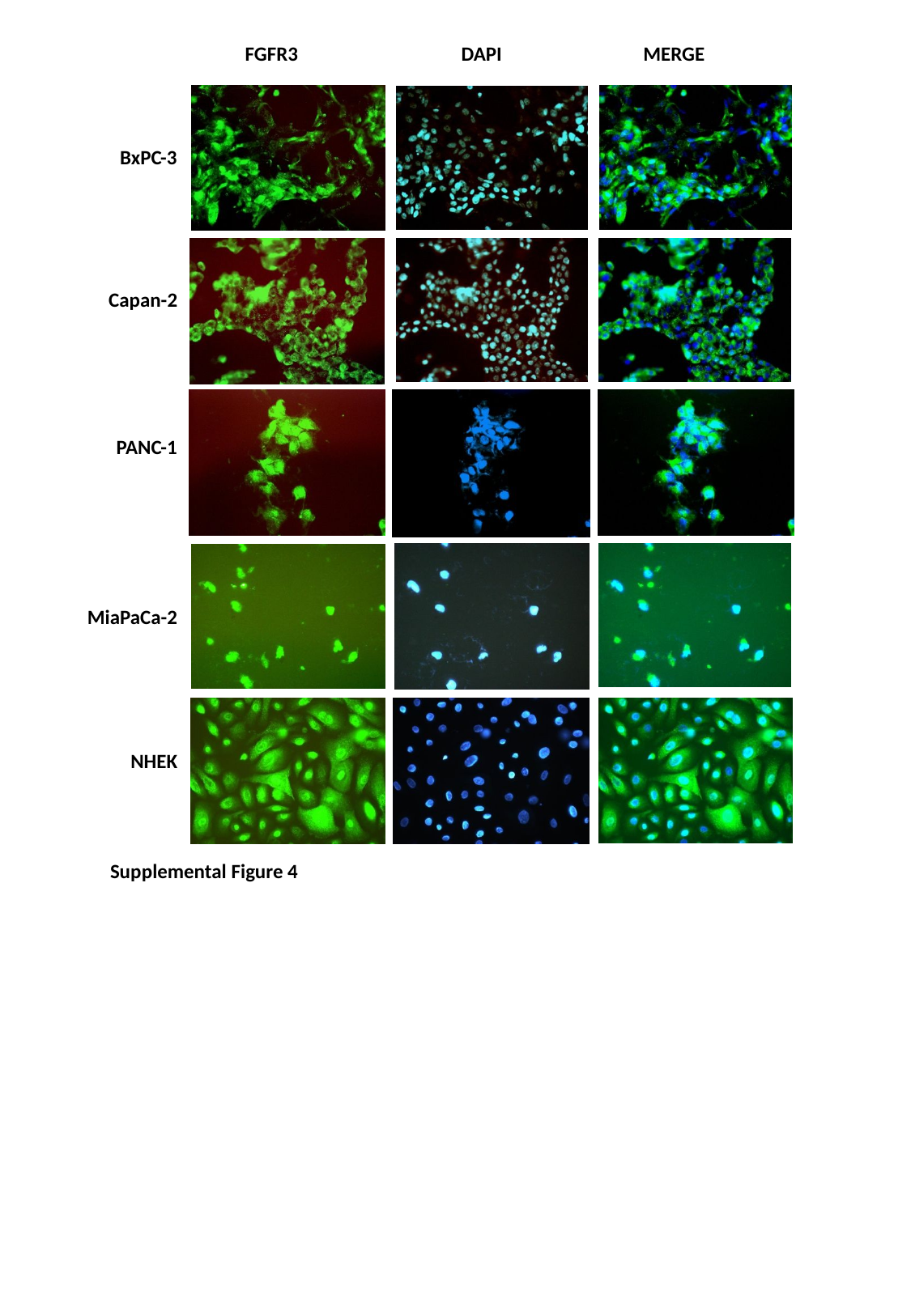

FGFR3
DAPI
MERGE
BxPC-3
Capan-2
PANC-1
MiaPaCa-2
NHEK
Supplemental Figure 4

Supplement: Additional file 4: Figure S4 — FGFR3 immuno-detection in pancreatic pareantal cell lines. Parental cell lines were culture on LABTEK chambers and FGFR3 presence was detected by immunofluorescence. Normal Human Epithelial Keratinocytes (NHEK) were used as positive controls for FGFR3 presence. Original magnification ×400. [file 1476-4598-12-83-S4.ppt]

## Slide 1
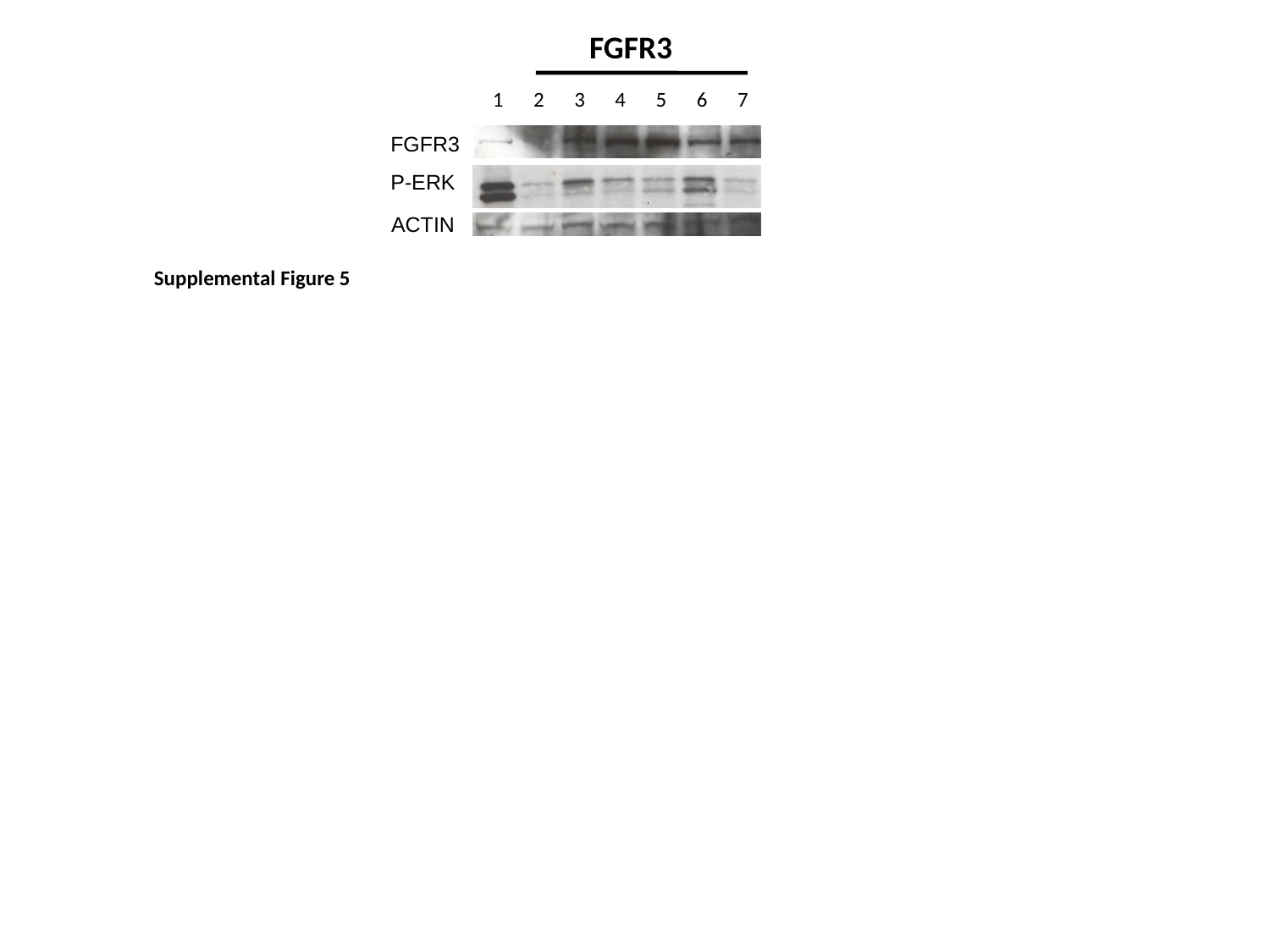

FGFR3
1
2
3
4
5
6
7
FGFR3
P-ERK
ACTIN
Supplemental Figure 5

Supplement: Additional file 5: Figure S5 — Western-blot of BTC cell extracts. Protein extracts from parental BTC line (lane 1) or from clones with FGFR3 overexpression (lane 2–7) have been subjected to western-blotting to detect FGFR3 and P-ERKs proteins levels. Actin protein was used as a loading control. [file 1476-4598-12-83-S5.pptx]
