# Supplementary material for: FGFR3 has tumor suppressor properties in cells with epithelial phenotype
Source: Mol Cancer. 2013 Jul 31;12:83. doi: 10.1186/1476-4598-12-83 (PMC3750311; doi:10.1186/1476-4598-12-83)
Supplement: Additional file 6: Figure S6 — FGF expression in pancreatic cancer cell lines. A) RT-qPCRs were performed as indicated in the materials and methods section to measure the levels of expression of different FGFR3 ligands in the parental cell lines. Primers sequences can be provided upon request. B) RT-qPCRs for FGF2 and FGF9 transcripts were performed on RNA extracts from cells transduced with FGFR3-IIIb and –IIIc variants or parental cells (CTRL). Results are reported according to the levels fround in CTRL. *: p < 0.05, **: p < 0.01, ***: p < 0.001 (n = 3, as compared to CTRL levels). Note that FGF9 was not detectable in MiaPaCa-2. [file 1476-4598-12-83-S6.pptx]

## Slide 1
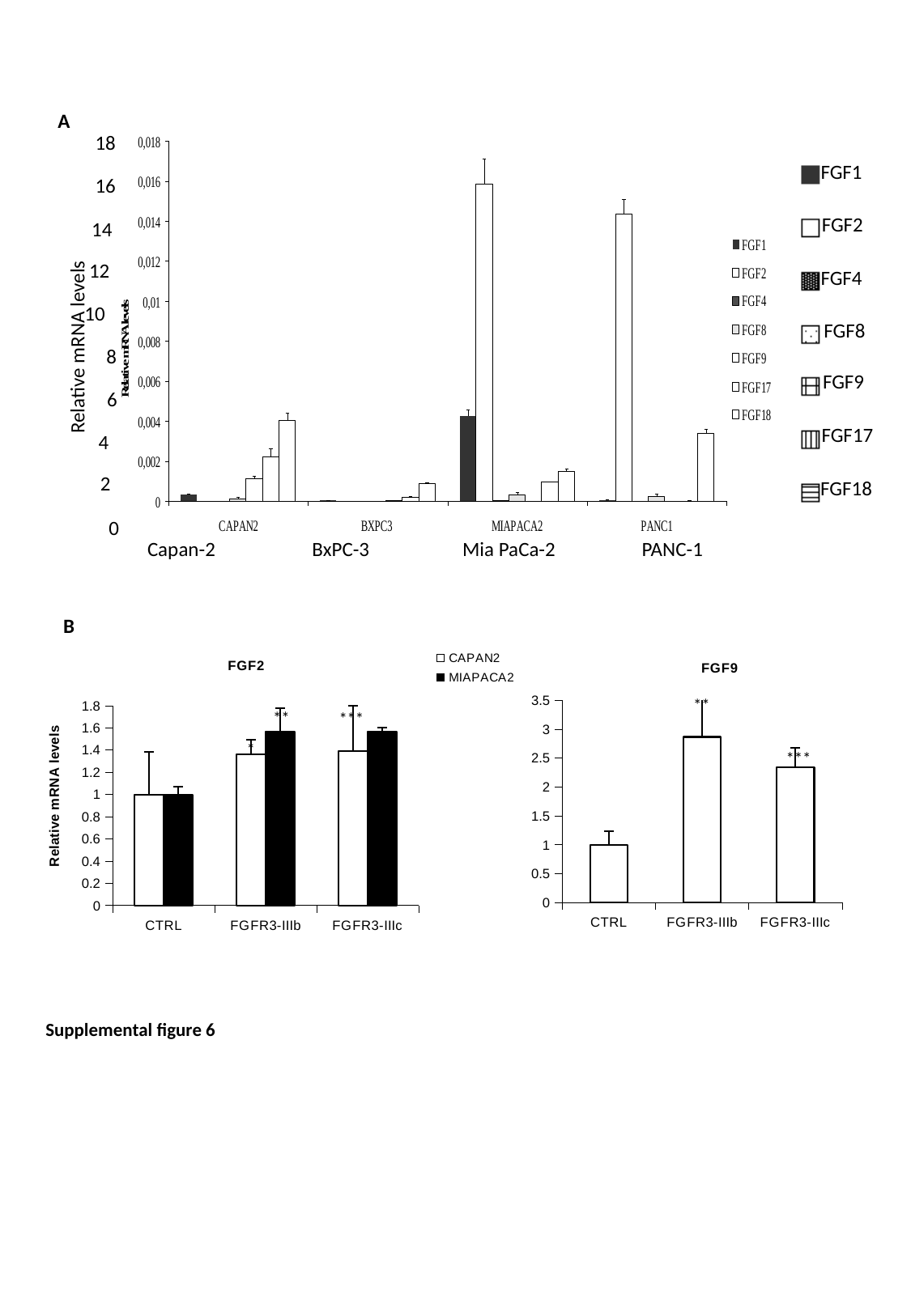

A
18
FGF1
FGF2
FGF4
FGF8
FGF9
FGF17
FGF18
16
14
12
Relative mRNA levels
10
8
6
4
2
0
Capan-2
BxPC-3
Mia PaCa-2
PANC-1
B
### Chart: FGF9
| Category | CAPAN2 |
|---|---|
| CTRL | 1.0 |
| FGFR3-IIIb | 2.864221778010835 |
| FGFR3-IIIc | 2.3417932489293807 |**
***
### Chart: FGF2
| Category | CAPAN2 | MIAPACA2 |
|---|---|---|
| CTRL | 1.0 | 1.0 |
| FGFR3-IIIb | 1.3636901074418022 | 1.5652533794122805 |
| FGFR3-IIIc | 1.3931241497525282 | 1.5634642743879397 |**
***
***
*
Supplemental figure 6
