# Supplementary material for: FGFR3 has tumor suppressor properties in cells with epithelial phenotype
Source: Mol Cancer. 2013 Jul 31;12:83. doi: 10.1186/1476-4598-12-83 (PMC3750311; doi:10.1186/1476-4598-12-83)
Supplement: Additional file 7: Figure S7 — Signaling pathways in BxPC-3 and PANC-1 tumor extracts. Proteins extracts of tumors from BxPc-3 and Mia PaCa-2, were analyzed by western-blot. See text for more details. CKIs: Cyclin-dependant kinase inhibitors. Membranes were reprobed for GAPDH to test equivalent loading. Results shown are representative of one out of at least 3 independent experiments. [file 1476-4598-12-83-S7.ppt]

## Slide 1
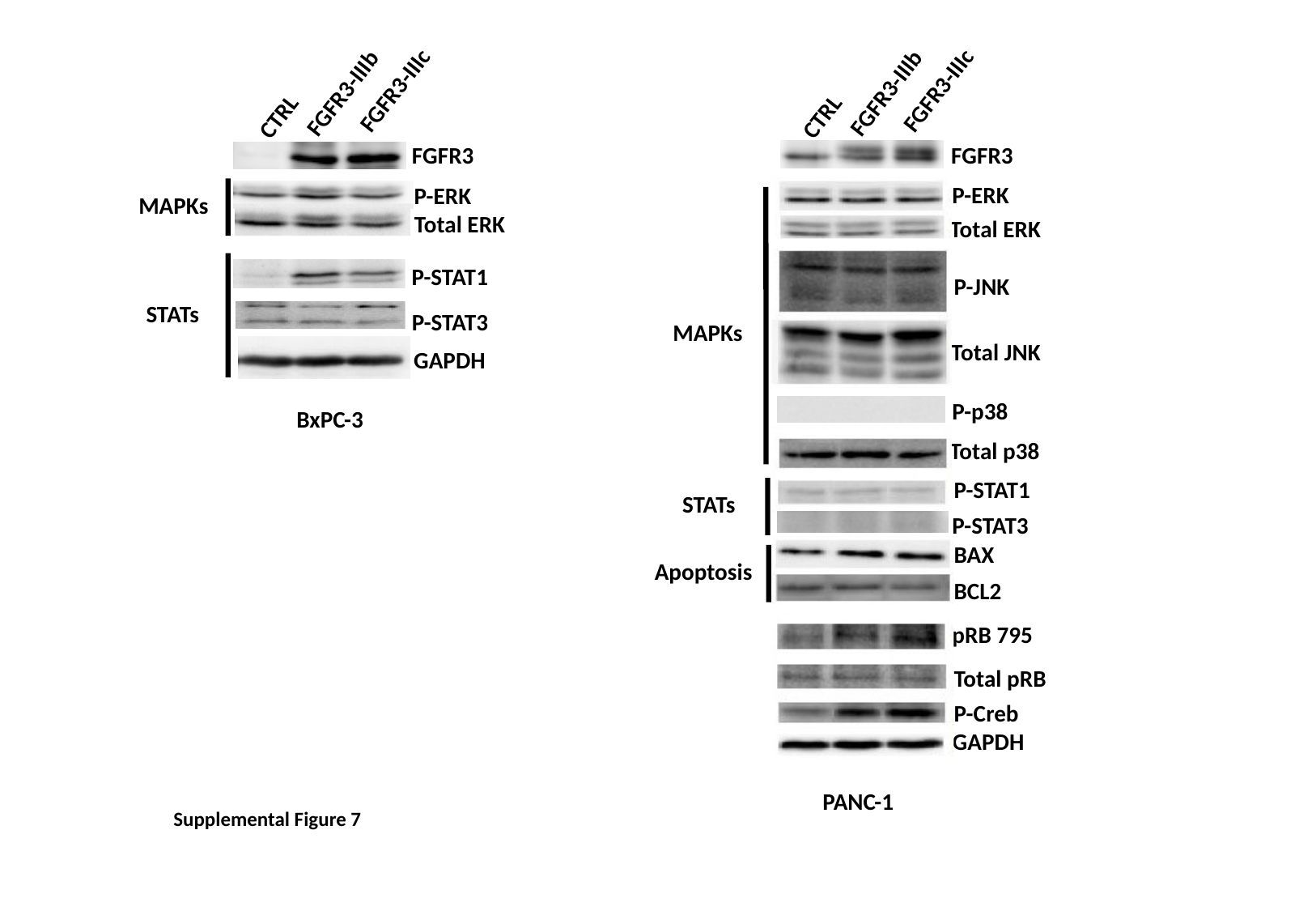

FGFR3-IIIc
FGFR3-IIIb
CTRL
FGFR3-IIIc
FGFR3-IIIb
CTRL
FGFR3
FGFR3
P-ERK
P-ERK
MAPKs
Total ERK
Total ERK
P-STAT1
P-STAT3
GAPDH
P-JNK
STATs
MAPKs
Total JNK
P-p38
BxPC-3
Total p38
P-STAT1
STATs
P-STAT3
BAX
Apoptosis
BCL2
P-pRB 795
Total pRB
P-Creb
GAPDH
PANC-1
Supplemental Figure 7
