# Supplementary material for: FGFR3 has tumor suppressor properties in cells with epithelial phenotype
Source: Mol Cancer. 2013 Jul 31;12:83. doi: 10.1186/1476-4598-12-83 (PMC3750311; doi:10.1186/1476-4598-12-83)
Supplement: Additional file 8: Table S1 — FGFR3 mRNA expression in normal pancreas and PDAC. [file 1476-4598-12-83-S8.docx]

**Table S1: FGFR3 mRNA expression in normal pancreas and PDAC**

|  | **Normal pancreas** | **PDAC** | **p value** |
| --- | --- | --- | --- |
| **FGFR3-IIIb** | 0.13±0.024 | 0.08±0.012 | 0.043 |
| **FGFR3-IIIc** | 0.17±0.024 | 0.07±0.015 | 0.005 |
| **N** | 10 | 29 |  |

RT-qPCRs were performed according to the description in materials and methods. Unpaired bilateral Student’s t tests were run to compare levels in normal pancreas compared to PDAC. The n numbers indicate the number of specimens in each group.
